# Hydroxyurea Mitigates Heme-Induced Inflammation and Kidney Injury in Humanized Sickle Cell Mice

**DOI:** 10.3390/ijms26073214

**Published:** 2025-03-30

**Authors:** William Kwaku Agbozo, Wesley Solomon, Cecilia Elorm Lekpor, Isaac Joe Erskine, Babayewa Oguljahan, Alaijah Bashi, Adriana Harbuzariu, Adel Driss, Samuel Adjei, Lily Paemka, Solomon Fifii Ofori-Acquah, Jonathan K. Stiles

**Affiliations:** 1 Department of Microbiology, Biochemistry and Immunology, Morehouse School of Medicine, Atlanta, GA 30310, USA; wagbozo@msm.edu (W.K.A.);; 2 Department of Biochemistry, Cell and Molecular Biology, College of Basic and Applied Sciences, University of Ghana, P.O. Box LG 25 Legon-Accra, Ghana; 3 West African Centre for Cell Biology of Infectious Pathogens (WACCBIP), College of Basic and Applied Sciences, University of Ghana, P.O. Box LG 25 Legon-Accra, Ghana; 4 Department of Pathology, Korle-Bu Teaching Hospital, P.O. Box 77 Korle Bu-Accra, Ghana; 5 Center for Laboratory Animal Resources, Morehouse School of Medicine, Atlanta, GA 30310, USA; 6 Stem Cell and Organoids Core, Emory University, Atlanta, GA 30322, USA; 7 Department of Animal Experimentation, Noguchi Memorial Institute for Medical Research, University of Ghana, P.O. Box LG 581 Legon-Accra, Ghana; 8 West African Genetic Medicine Center (WAGMC), University of Ghana, P.O. Box LG 25 Legon-Accra, Ghana

**Keywords:** hydroxyurea, sickle cell disease, acute kidney injury, heme, inflammation

## Abstract

Kidney disorders significantly contribute to morbidity and mortality in sickle cell disease (SCD). Acute kidney injury (AKI), a major risk factor for chronic kidney disease (CKD), often arises from intravascular hemolysis, where plasma cell-free heme drives AKI through inflammatory and oxidative stress mechanisms. Hydroxyurea (HU), a well-established SCD-modifying therapy, improves clinical outcomes, but its effects on systemic heme and inflammatory mediators of kidney injury remain underexplored. This study evaluated HU’s impact on plasma heme, pro-inflammatory mediators, kidney injury, and renal histopathology in a sickle cell mouse model. Townes humanized sickle cell mice (HbSS) and non-sickle (HbAA) controls were treated with HU or vehicle for two weeks. HU significantly reduced total plasma heme, lactate dehydrogenase, and pro-inflammatory cytokines (CXCL10, VEGF-A, IFN-γ) in HbSS mice. HU reduced renal injury biomarkers (cystatin C, NGAL) and improved renal histopathology, evidenced by reduced vascular congestion, glomerulosclerosis, and tubular damage. Interestingly, HU did not alter the levels of kidney repair biomarkers (clusterin and EGF). These findings suggest that HU mitigates kidney injury by reducing the deleterious effects of circulating heme and inflammation, supporting its potential to slow or prevent progressive kidney injury in SCD.

## 1. Introduction

Sickle cell disease (SCD) is an inherited blood disorder characterized by chronic hemolysis, vaso-occlusion, and systemic inflammation, which together drive multiorgan damage, including significant renal pathology [1,2]. Acute kidney injury (AKI), defined as the sudden loss of kidney function, is particularly concerning due to its prevalence among hospitalized SCD patients and is associated with the progressive deterioration of renal health, resulting in chronic kidney disease (CKD) [3,4]. CKD affects over half of SCD patients by age 40 years and is responsible for up to 16–18% of SCD-related deaths [5].

Acute elevations in circulating cell-free heme during intravascular hemolysis is implicated in the development of SCD-related AKI [6,7]. Circulating cell-free heme, a potent pro-oxidant, induces systemic and local inflammation as well as oxidative stress, contributing to glomerular endothelial injury [8]. When exposed to the kidney, cell-free heme initiates cytotoxic processes that result in renal tubular dysfunction and glomerular damage [6,9]. Additionally, chronically elevated inflammatory chemokines and cytokines further contribute to kidney injury and impaired kidney function in SCD [10,11,12]. Suggested mechanisms of kidney protection in SCD include reducing plasma heme levels, reactive oxygen species, and inflammatory mediators. However, the extent to which current therapies effectively modulate these factors and prevent progressive kidney injury in SCD remains only partially understood.

Hydroxyurea (HU), a ribonucleotide reductase inhibitor, is the standard disease-modifying therapy for SCD [13]. By increasing fetal hemoglobin, reducing white blood cell and platelet counts, and enhancing nitric oxide bioavailability, HU decreases erythrocyte sickling and mitigates vaso-occlusion, hemolysis, and endothelial injury [14]. Clinically, HU reduces stroke risk and prevents cardiac, respiratory, and splenic dysfunction and mortality [15]. Additionally, HU improves renal outcomes, including reductions in albuminuria and serum creatinine in patients with SCD [15,16,17]. Studies by Park et al. (2019) have in part demonstrated that there may be a benefit to HU administration in mitigating renal injury, particularly following ischemia–reperfusion renal injury [18]. However, HU’s impact on hemolysis-driven kidney injury and related histological changes in SCD remains inadequately characterized and needs to be further evaluated. Additionally, HU is recognized for its potential anti-inflammatory and antioxidant properties [19,20,21,22,23]. Importantly, the effect of hydroxyurea on key inflammatory cytokines/chemokines implicated in SCD-associated kidney injury, such as IP-10/CXCL10, MCP-1/CCL2, and IL-6 [10,12] remains insufficiently investigated.

This study investigated the effect of HU on circulating heme and pro-inflammatory mediators, biomarkers of kidney injury and repair, and histopathologic renal damage in the HbSS-Townes mouse, a model that closely mimics human SCD-associated kidney pathology [24,25,26].

## 2. Results

### 2.1. Hydroxyurea Modulates Markers of Systemic Hemolysis and Inflammation Relevant to Kidney Injury in Townes Humanized Sickle Cell Mice

**Figure 1:**
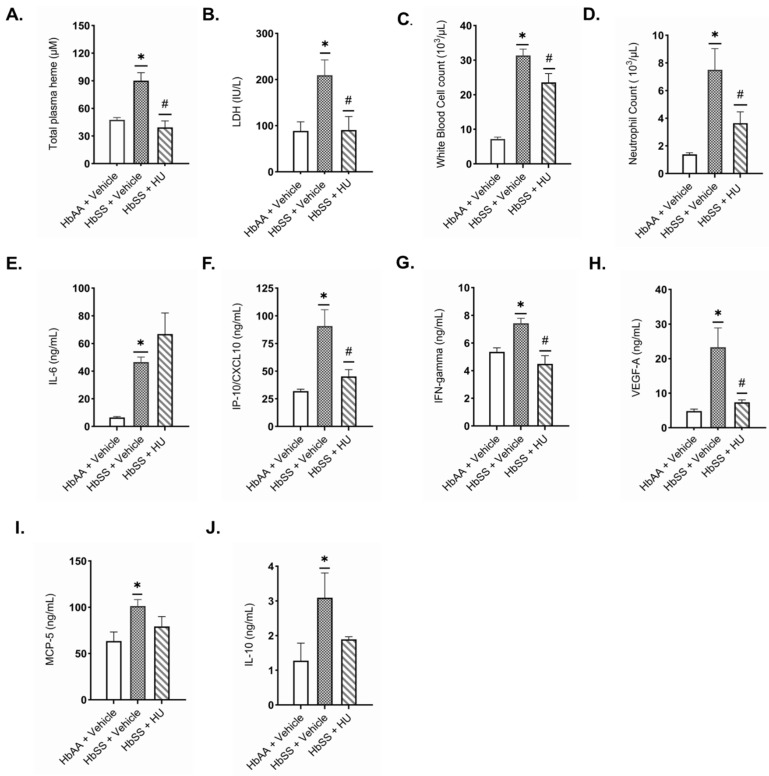
Hydroxyurea modulates markers of systemic hemolysis and inflammation relevant to kidney injury in Townes humanized sickle cell mice. Plasma levels of hemolysis markers: (**A**) total plasma heme and (**B**) lactate dehydrogenase; (**C**) white blood cell count; (**D**) neutrophil count, and serum pro-inflammatory markers: (**E**) interleukin-6 (IL-6); (**F**) C-X-C motif chemokine ligand 10 (CXCL10); (**G**) interferon-gamma (IFN-γ); (**H**) vascular endothelial growth factor-A (VEGF-A); (**I**) murine monocyte chemoattractant protein-5 (MCP-5); and anti-inflammatory marker: (**J**) interleukin-10 (IL-10) were assessed in 12-week-old Townes mice after two weeks of treatment with either the vehicle or hydroxyurea (50 mg/kg/day) (n = 7–8 per group). Data are presented as the mean ± SEM. HbAA indicates the genetic control mice; HbSS, SCD mice. * *p* < 0.05 vs. HbAA vehicle; ^#^ *p* < 0.05 vs. HbSS vehicle.

Evidence of a hyperhemolytic state was observed in vehicle-treated sickle cell mice (HbSS) with high levels of total plasma heme and lactate dehydrogenase (LDH) activity compared to the controls (HbAA) (Figure 1A,B). Hydroxyurea (HU) treatment significantly reduced total plasma heme (39.3 ± 7.1 µM vs. 90.2 ± 8.7 µM, *p* = 0.001) and LDH levels (90.6 ± 29.4 IU/L vs. 209.3 ± 33.3 IU/L, *p* = 0.023) in HbSS mice compared to vehicle-treated HbSS controls (Figure 1A,B). Circulating pro-inflammatory cytokines relevant to kidney injury were also elevated in HbSS mice, including interleukin-6 (IL-6), CXCL10 (IP-10), interferon-gamma (IFN-γ), vascular endothelial growth factor-A (VEGF-A), and murine monocyte chemoattractant protein-5 (MCP-5), compared to HbAA controls. In part, the HU treatment demonstrated anti-inflammatory effects in HbSS mice by showing a significant reduction in WBC counts (22.9 ± 1.8 × 10^3^/μL vs. 31.4 ± 1.8 × 10^3^/μL, *p* = 0.012), neutrophil counts (4.0 ± 0.6 × 10^3^/µL vs. 7.5 ± 1.5 × 10^3^/μL, *p* = 0.013), and serum levels of CXCL10 (45.2 ± 6.2 ng/mL vs. 90.7 ± 14.9 ng/mL, *p* = 0.003), IFN-γ (4.5 ± 0.6 ng/mL vs. 7.4 ± 0.4 ng/mL, *p* = 0.013), and VEGF-A (7.3 ± 0.7 ng/mL vs. 23.3 ± 5.6 ng/mL, *p* = 0.003) versus vehicle-treated HbSS mice (Figure 1C–H). While MCP-5 levels decreased, the reduction was not statistically significant. IL-6 levels remained elevated post-HU treatment (Figure 1E). Interleukin 10 (IL-10), an anti-inflammatory marker, was significantly higher in vehicle-treated HbSS mice compared to HbAA controls, with no significant change observed with HU treatment (Figure 1J). These findings show HU potential to reduce circulating heme and inflammatory cytokines, factors that mediate kidney injury in SCD.

### 2.2. Hydroxyurea Treatment Mitigates Kidney Injury in Townes Humanized Sickle Cell Mice

**Figure 2:**
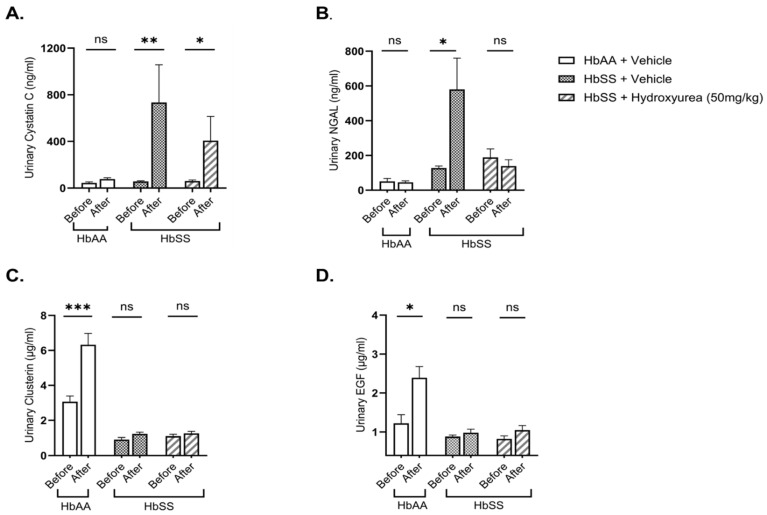
Hydroxyurea mitigates kidney injury in Townes humanized sickle cell mice. Urinary levels of kidney injury biomarkers: (**A**) cystatin C and (**B**) neutrophil gelatinase-associated lipocalin (NGAL) and kidney repair biomarkers (**C**) clusterin and (**D**) epidermal growth factor (EGF) were measured in 12-week-old sickle cell (HbSS) mice and genetic control (HbAA) mice (n = 7–8 per group) and after 2 weeks of treatment with hydroxyurea (50 mg/kg/day) or the vehicle. Data are presented as the mean ± SEM. Changes in biomarker levels pre- and post-treatment were analyzed using paired ratio *t*-tests (two-tailed). ns: not significant; *p* < 0.05 (*); *p* < 0.01 (**); *p* < 0.001 (***).

Kidney injury biomarkers—cystatin C, neutrophil gelatinase-associated lipocalin (NGAL) and kidney repair biomarkers (clusterin, epidermal growth factor (EGF)—were assessed in urine before and after two weeks of treatment. After the experimental period, the HbSS vehicle-treated group had a significant increase in cystatin C (12.3-fold, *p* = 0.006) and NGAL (4.1-fold, *p* = 0.015) relative to their baseline levels, whereas the HbAA control group showed no significant changes (Figure 2A,B). HU treatment mitigated this increase, resulting in a lower pre- and post-treatment fold change in cystatin C (8.6-fold, *p* = 0.039) compared to the HbSS vehicle-treated group (12.3-fold, *p* = 0.006) (Figure 2A). Notably, NGAL levels remained unchanged over the experimental period in theHbSS HU-treated group, in contrast to the significant 4.1-fold increase (*p* = 0.015) in the HbSS vehicle-treated group (Figure 2B).

For renal repair markers, post-treatment urinary clusterin (1.2 ± 0.1 µg/mL vs. 6.3 ± 0.6 µg/mL, *p* = 0.001) and EGF (1.0 ± 0.1 µg/mL vs. 2.8 ± 0.1 µg/mL, *p* = 0.029) levels were significantly reduced in the HbSS vehicle-treated group compared to the HbAA controls. Clusterin and EGF levels significantly increased from baseline in the HbAA vehicle-treated group but remained unchanged in the HbSS vehicle-treated group. HU treatment did not significantly alter urine clusterin or EGF levels in HbSS mice (Figure 2C,D). These findings suggest that while HU attenuates kidney injury severity in SCD, it does not appear to do so via upregulation of renal repair factors.

### 2.3. Hydroxyurea Ameliorates Renal Histopathologic Changes in Townes Humanized Sickle Cell Mice

**Figure 3:**
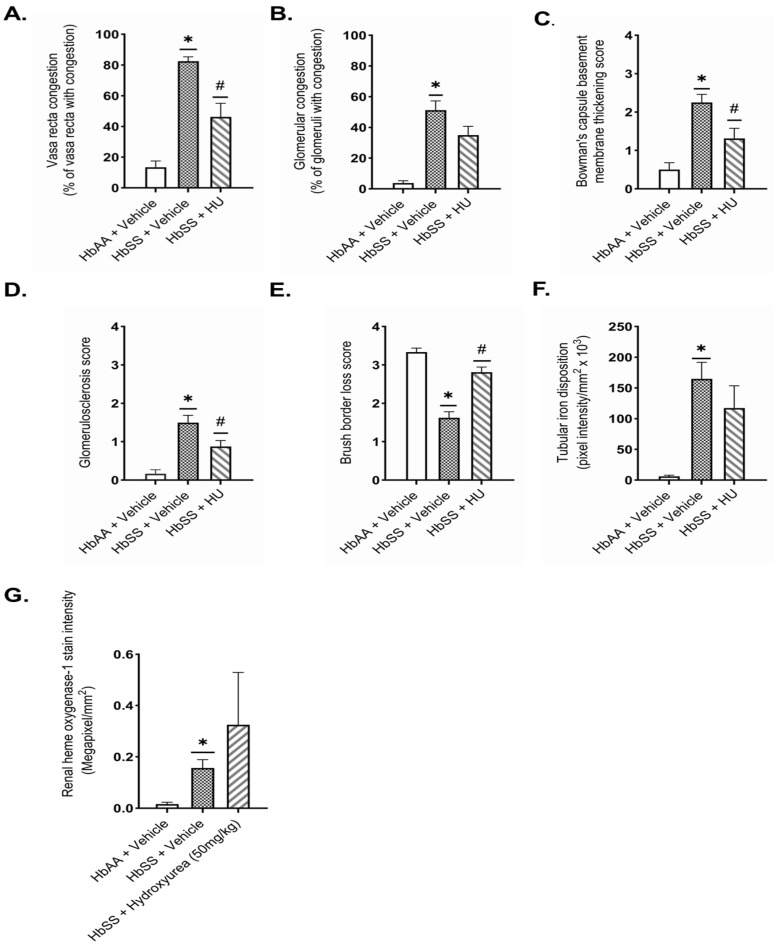

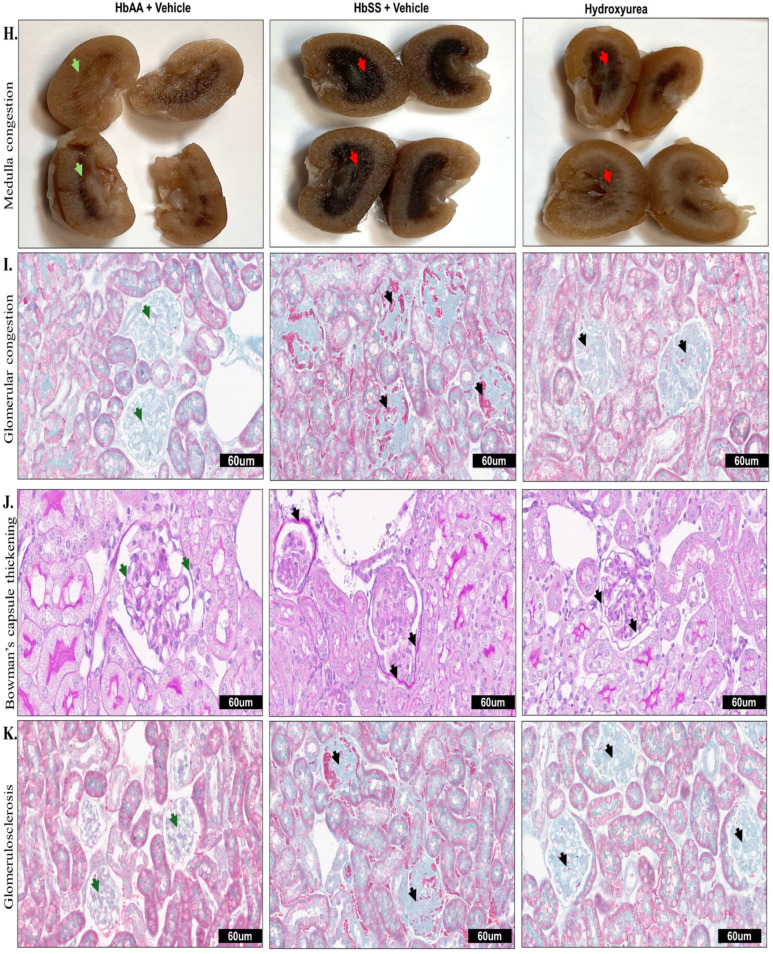

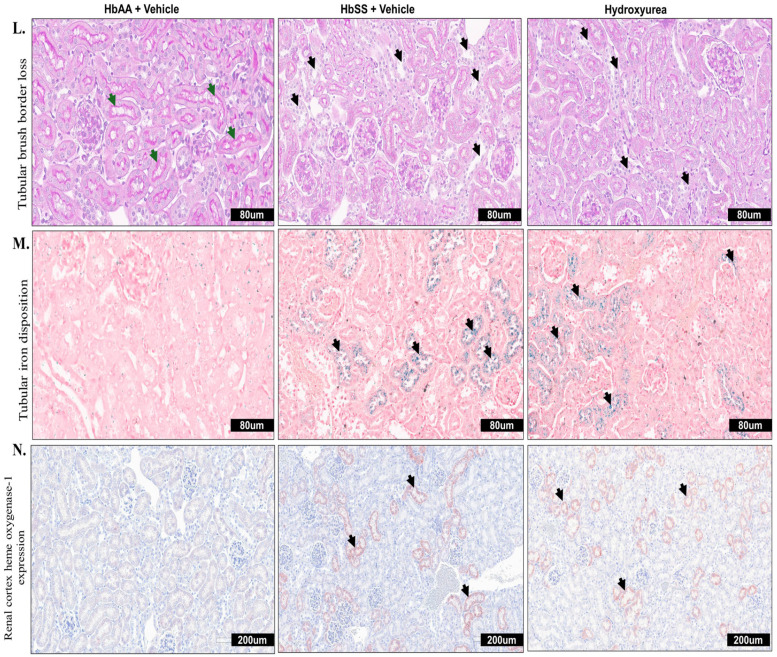
Hydroxyurea attenuates kidney damage in Townes humanized sickle cell mice. Histological analysis of renal tissues from sickle cell (HbSS) and non-sickle control (HbAA) mice following vehicle or hydroxyurea (50 mg/kg/day) treatment for 2 weeks at 12 weeks of age (n = 7–8 per group). Quantification of (**A**) vasa recta congestion, (**B**) glomerular congestion, (**C**) Bowman’s capsule thickening, (**D**) glomerulosclerosis, (**E**) tubular brush border loss, (**F**) intensity of tubular iron deposition, and (**G**) kidneyl heme oxygenase-1 expression. Data represent the mean ± SEM. * *p* < 0.05 vs. HbAA vehicle; ^#^ *p* < 0.05 vs. HbSS vehicle. Panel (**H**) shows halved kidneys with mild medulla congestion in a vehicle-treated HbAA mouse (green arrowheads), severe medulla congestion in a vehicle-treated HbSS mouse, and mild congestion in an HU-treated HbSS mouse (red arrowheads). Panel (**I**) shows representative Masson trichrome–stained sections, highlighting normal, uncongested glomeruli in a vehicle-treated HbAA mouse (green arrowheads), severe congestion in a vehicle-treated HbSS mouse, and moderate congestion in an HU-treated HbSS mouse (black arrowheads). Panel (**J**) depicts representative PAS-stained sections, showing a normal Bowman’s capsule membrane in a vehicle-treated HbAA mouse (green arrowheads), moderate thickening in a vehicle-treated HbSS mouse, and mild thickening in an HU-treated HbSS mouse (black arrowheads). Panel (**K**) shows Masson trichrome–stained sections, indicating normal glomeruli with no sclerosis in a vehicle-treated HbAA mouse (green arrowheads), moderate glomerulosclerosis in a vehicle-treated HbSS mouse, and mild glomerulosclerosis in an HU-treated HbSS mouse (black arrowheads). Panel (**L**) shows PAS-stained sections of cortical tubules, with a stained epithelial cell brush border in a vehicle-treated HbAA mouse (green arrowheads), severe tubular brush border loss in a vehicle-treated HbSS mouse, and mild tubular brush border loss in an HU-treated HbSS mouse (black arrowheads). Panel (**M**) shows Perl’s Prussian blue–stained renal cortex sections, demonstrating no tubular iron deposits in a vehicle-treated HbAA mouse and iron deposits in a vehicle-treated and HU-treated HbSS mouse (black arrowheads). Panel (**N**) shows immunohistochemistry staining of anti-heme oxygenase-1 in cortex sections with nearly absent HO-1 expression in a vehicle-treated HbAA mouse and mild expression in both an HU- and vehicle-treated sickle cell mouse (black arrowheads). Original magnification, ×40.

Hydroxyurea treatment improved renal histopathologic changes associated with kidney injury in sickle cell mice (HbSS) following two weeks of treatment. Vehicle-treated HbSS mice exhibited marked renal pathology compared to HbAA controls, characterized by pronounced medullary and glomerular vascular congestion, Bowman’s capsule thickening, glomerulosclerosis (>25% of glomeruli affected), and cortical tubular injury with brush border loss (>50% of tubules affected) (Figure 3). HU treatment significantly improved these histopathological changes. HU-treated mice showed reduced vasa recta congestion (46.3 ± 8.8 score vs. 82.5 ± 2.8 score, *p* = 0.002) (Figure 3A,H) and moderate improvement in Bowman’s capsule thickening (1.3 ± 0.3 score vs. 2.3 ± 0.2 score, *p* = 0.037) (Figure 3C,J). Glomerulosclerosis was markedly reduced, with <25% of glomeruli exhibiting mild sclerosis (0.9 ± 0.2 score vs. 1.5 ± 0.2 score, *p* = 0.014) (Figure 3D,K). Additionally, HU improved the extent of tubular brush border loss compared to vehicle-treated sickle cell mice (2.8 ± 0.1 score vs. 1.6 ± 0.2 score, *p* ˂ 0.001) (Figure 3E,L). Iron accumulation within cortical tubules was observed in both treated and untreated HbSS mice, with a modest, non-significant reduction in HU-treated mice (Figure 3F,M). HU treatment did not significantly alter heme oxygenase-1 expression (0.5 ± 0.3 vs. 0.2 ± 0.04 megapixels/mm^2^, *p* = 0.755) compared to vehicle-treated HbSS mice (Figure 3G,N). These findings underscore hydroxyurea’s potential to mitigate the renal histopathological changes associated with SCD, notably glomerulosclerosis and vascular congestion.

## 3. Discussion

This study highlights the therapeutic potential of hydroxyurea (HU) in mitigating renal injury in a humanized mouse model of sickle cell disease (SCD). Our findings provide insights into HU’s ability to reduce circulating heme, pro-inflammatory cytokines associated with SCD-kidney injury, thereby ameliorating the renal injury and damage in SCD mice.

As shown in prior studies, untreated sickle cell (HbSS) mice exhibited an elevated plasma heme and LDH levels, consistent with the well-documentedexcess circulating heme in SCD that drives oxidative stress, endothelial dysfunction, and organ damage [1]. Our study demonstrates that the HU treatment significantly reduced total plasma heme and LDH activity in SCD mice. This effect likely stems from HU’s ability to stabilize erythrocytes through multiple mechanisms: increasing fetal hemoglobin (HbF) production, reducing sickle hemoglobin polymerization, and improving red cell hydration, all of which contribute to decreased erythrocyte breakdown [13,27]. The significance of these findings is supported by prior human and mouse studies demonstrating strong associations between elevated plasma LDH or heme levels and declining kidney function [17,28,29,30,31,32]. By reducing plasma heme, HU treatment may effectively mitigate the heme-driven cascade of renal inflammation, oxidative stress, and subsequent kidney injury.

The chronic inflammatory state in SCD, characterized by leukocytosis and elevated pro-inflammatory cytokines, correlates with deteriorating kidney function [10]. In both human and animal models, HU therapy has been shown to significantly reduce elevated inflammatory cytokines [19,20,23]. Consistent with these findings, our results show that HU reduces WBC and neutrophil counts, as well as pro-inflammatory cytokines CXCL10, VEGF-A, and IFN-γ. While Zahran et al. reported a significant decrease in the serum IL-6 levels of SCD patients treated with HU [23], other studies have found no significant reduction, including our findings [22,33]. Given IL-6’s role in inflammation and kidney disease, the conflicting findings on HU’s effect warrant further studies to define its impact on IL-6 regulation in SCD-related kidney injury.

Sickle cell mice showed signs of kidney injury, with elevated urinary levels of cystatin C and NGAL. These findings agree with the extensive literature identifying acute kidney injury as a common and persistent complication in SCD, driven by hemolysis, ischemia–reperfusion injury, and chronic inflammation [3,4,6,10,18,34,35,36]. Park et al. (2019) showed that HU significantly lowered serum creatinine, NGAL, and KIM-1 levels in an ischemia–reperfusion injury model [18]. Consistent with these findings, our study confirms that HU reduces urinary cystatin C and NGAL, further supporting its renoprotective effects in SCD. The precise mechanism by which HU mitigates kidney injury remains unclear, but prior studies suggest it is indirectly linked to reduced hemolysis, a hypothesis supported by our results [6,17,29,30,31,32].

Although HU effectively reduces kidney injury severity, it did not alter the urinary levels of clusterin and epidermal growth factor (EGF), biomarkers of renal repair. Clusterin has been implicated in tubular protection and recovery in kidney injury models, while reduced urinary EGF is linked to impaired renal injury repair and worsened kidney disease outcomes [37,38,39,40,41,42]. Our findings suggest that HU’s protective effects may be mediated primarily through injury reduction rather than direct stimulation of intrinsic repair pathways. Further studies are needed to explore potential therapeutic strategies for enhancing kidney repair in SCD-associated nephropathy.

Histological analysis supported the observation that HU mitigates kidney injury in our study. HU-treated HbSS mice exhibited reduced glomerulosclerosis, mild vasa recta congestion, and preserved cortical brush border integrity, reinforcing HU’s protective role in maintaining glomerular and renal tubular structures. Consistent with prior studies, these findings further confirm HU’s renoprotective effects in SCD [18,43]. Previous studies have shown that HU increases nitric oxide bioavailability, reduces endothelial adhesion molecule expression, and decreases leukocyte adhesion [19,44,45,46], mechanisms that may contribute to its effect on renal vascular congestion. The reduction in glomerulosclerosis, a hallmark of progressive kidney disease, aligns with clinical studies showing that HU improves markers of glomerulopathy, such as albuminuria and glomerular filtration rates, further supporting its renoprotective potential [16,17,47,48]. Acute hemolysis increases renal tubular uptake of heme, leading to heme-dependent iron overload, oxidative stress, ferroptosis, and mitochondrial dysfunction [49,50,51]. Taylor et al. [43] previously reported that short-term HU treatment does not significantly alter renal iron accumulation in sickle cell mice, consistent with our findings of only modest and statistically nonsignificant reductions in cortical tubular iron deposition. However, clinical evidence suggests that prolonged HU therapy may reverse renal iron accumulation in patients [52], highlighting the importance of further investigation into HU’s long-term renal effects. The protective role of heme oxygenase-1 (HO-1) against heme-mediated kidney injury is well-established [53,54,55]. In our study, although HU treatment appears to increase HO-1 expression in HbSS mice, it was statistically not significant. Previous in vitro studies show that HU did not modulate HO-1 expression in human peripheral blood mononuclear cells or endothelial cells, following heme exposure [21]. While this study did not assess heme content in the kidneys, it is possible that hydroxyurea-mediated kidney HO-1 induction may be a possible mechanism in mitigating heme-induced kidney injury. Given HO-1’s essential role in renal protection, further studies exploring HU’s potential impact on HO-1 expression and other heme scavengers are warranted.

In conclusion, our findings highlight hydroxyurea’s therapeutic potential to attenuate kidney injury, a major contributor to SCD-related morbidity and mortality [4,35,56]. Using a preclinical mouse model of SCD, we have demonstrated that HU effectively reduces the deleterious effect of heme-induced inflammation, and kidney injury while preserving renal architecture by attenuating glomerulosclerosis, vascular congestion, and cortical tubular brush border loss. Although HU was administered for a short-term duration, the accelerated disease progression in murine SCD models facilitates the early detection of biologically meaningful changes within this short period of time. Moreover, short-term HU treatment in mice approximates prolonged exposure in humans, underscoring the translational relevance of our findings [57]. Utilizing 12-week-old Townes sickle cell mice—an age at which kidney injury first becomes evident [26]—our results highlight HU’s potential to slow or prevent the progression of renal injury in SCD, further supporting its continued clinical application.

## 4. Materials and Methods

### 4.1. Animals

All animal procedures were conducted in compliance with the National Institutes of Health Guide for the Care and Use of Laboratory Animals and were approved by the Institutional Animal Care and Use Committee (IACUC) (Protocol # 22-14) at Morehouse School of Medicine, Atlanta, GA, USA. Sex-matched humanized Townes (*HbSS*) sickle cell mice and their non-sickle genetic controls (*HbAA*), engineered by replacing endogenous mouse α- and β-globin genes with their human counterparts, ref. [24] were obtained from the Jackson Laboratory (Bar Harbor, ME, USA; JAX stock #01307). The Townes sickle cell mice (*Hba hα/hα::Hbb hAγβ^S^/hAγβ^S^)* express human hemoglobin S (HbS), mimicking the severe hemolytic anemia and kidney pathology characteristic of human SCD [25,26]. Mice were maintained in a controlled environment with regulated temperature, humidity, a 12 h light/dark cycle, and ad libitum access to a standard rodent diet (PMI LabDiet 5001) and water.

### 4.2. Study Design

A cohort of sex-matched Townes mice aged twelve-weeks old were randomized into three experimental groups (n = 7–8 per group): (1) HbAA + vehicle; (2) HbSS + vehicle; (3) HbSS + hydroxyurea (HU). At 12-weeks old, Townes mice spontaneously develop significant tubular and glomerular injury [26,43,58]. Similar to HU treatment regimens in humans [59], we used a 50 mg/kg/day HU dose that has been shown to be well-tolerated, safer, and effective in inducing significant hemoglobin F levels in the Townes sickle cell model [60,61,62], with reported renal protective effects [18,43,44]. Mice were treated with 50 mg/kg/day HU (Sigma-Aldrich, St. Louis, MO, USA. SKU # H8627) five times per week for two weeks via intraperitoneal injection. Hydroxyurea was freshly prepared in sterile phosphate-buffered saline (PBS; R&D Biosystems, Minneapolis, MN, USA), filtered through a 0.2 μm syringe filter and administered in a 50 μL dose. Control animals received 50 μL of sterile PBS. At the end of the study, urine, blood, and kidney samples were collected for analysis.

### 4.3. Blood and Urine Analyses

Urine samples were collected pre- and post-treatment following Chew and Chua’s protocol [63]. Blood was obtained via cardiac puncture, processed, aliquoted, and stored at −80 °C. Urinary kidney injury and repair biomarkers were measured using the Milliplex Mouse Kidney Injury Magnetic Bead Kit (Millipore Sigma, St. Louis, MO, USA) per the manufacturer’s instructions. White blood counts were analyzed using an automated veterinary hematology analyzer Element HT5 (Heska, Loveland, CO, USA). Total plasma heme and lactate dehydrogenase (LDH) levels were assessed using colorimetric kits (Bioassay Systems, Hayward, CA, USA). Serum inflammatory cytokines were quantified with the Milliplex Mouse Cytokine/Chemokine Magnetic Bead Kit (Millipore Sigma, St. Louis, MO, USA).

### 4.4. Histology

Kidneys from HbSS and HbAA mice were fixed in 10% formalin for 16–18 h and paraffin-embedded. Tissue sections (4 μm) were stained with hematoxylin and eosin, Masson’s trichrome, periodic acid–Schiff (PAS), and Perls’ Prussian blue. Histological examination and scoring were performed blinded using established criteria for renal structural changes [26,58]. Tubular iron accumulation was quantified by measuring blue-stained areas in the cortex using Aperio ImageScope positive pixel count v9 software (Leica Biosystems, Deer Park, IL, USA; version 12.4.6.5003) and normalized to the annotated region area (pixel intensity/mm^2^). Immunohistochemistry was performed using an Autostainer 360 (Epredia, Kalamazoo, MI, USA). Tissue sections underwent deparaffinization, rehydration, and antigen retrieval in a PT Module (Epredia, Kalamazoo, MI, USA) with Dewax and HIER Buffer H (pH 8.6–9.0) (Epredia, Kalamazoo, MI, USA. CAT # TA-999-DHBH). Sections were blocked and incubated with primary antibody anti-heme oxygenase-1 (1:200) (Abcam Inc., Waltham, MA, USA. CAT # ab52947). AEC chromogen was used for detection, followed by hematoxylin counterstaining and aqueous mounting. Slides were scanned at 40× magnification, and staining was quantified using the Aperio ImageScope positive pixel count algorithm (Leica Biosystems, Deer Park, IL, USA; version 12.4.6.5003). Negative controls were used to set thresholds, and data were normalized to annotated tissue area. Results were averaged per group.

### 4.5. Statistics

Statistical analyses were performed using GraphPad Prism v10.4.1. Two-group comparisons used two-tailed, unpaired t-tests, while a one-way ANOVA with a Tukey’s post hoc test assessed differences among groups. Paired ratio t-tests evaluated pre- and post-treatment urine biomarker changes. Results are expressed as the means ± standard error of the mean (SEM), with *p* < 0.05 being considered statistically significant.

## Data Availability

All relevant data are within the manuscript.
